# Supplementary material for: Genome-wide CRISPR screens identify GATA6 as a proviral host factor for SARS-CoV-2 via modulation of ACE2
Source: Nat Commun. 2022 Apr 25;13:2237. doi: 10.1038/s41467-022-29896-z (PMC9039069; doi:10.1038/s41467-022-29896-z)

Supplementary Information

Genome-wide CRISPR screens identify GATA6 as a proviral host factor for SARS-CoV-2 via modulation of ACE2

M. Israeli, Y. Finkel *et al*

Supplementary Figure 1

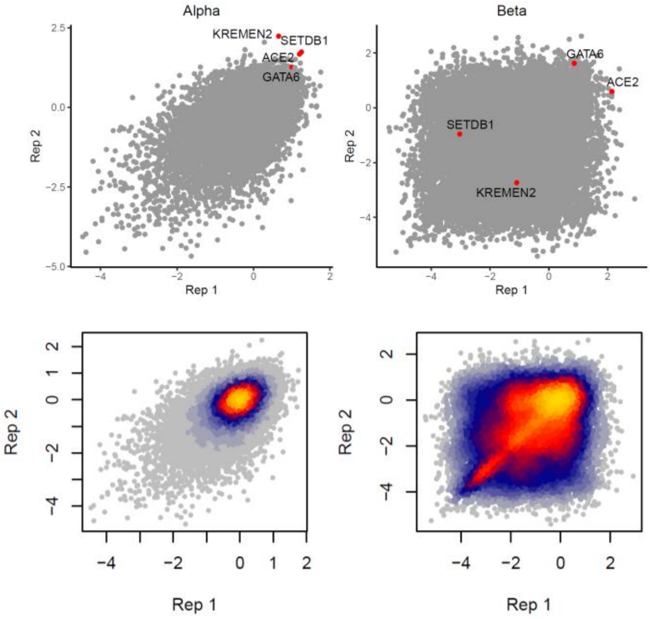

Supplementary Figure 1. Reproducibility of the log fold change (LFC) of sgRNAs between replicates of the screens of Alpha and Beta VOCs. Top pro-viral candidates identified in our screens are displayed.

<u>Supplementary Figure 2</u>

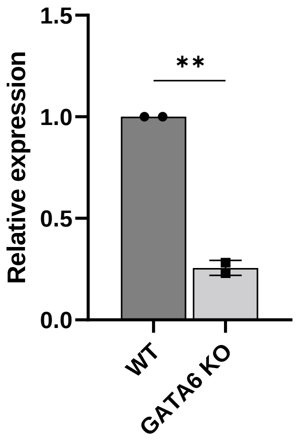

Supplementary Figure 2. real-time PCR measurements of GATA6 mRNA levels relative to GAPDH amount in Control and GATA6 disrupted Vero-E6 cells.

Supplementary Figure 3

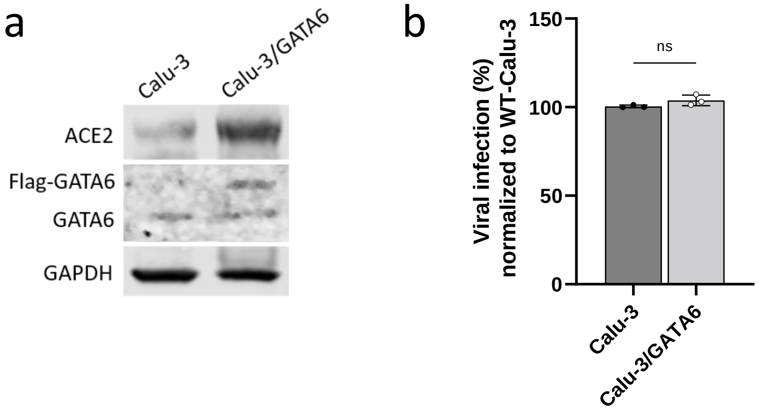

Supplementary Figure 3. (a) Western blot for the detection of GATA6 and ACE2 levels was performed in control and GATA6-over expressed Calu-3 cells. **(b)** real-time PCR quantification of WT-SARS-CoV-2 in control and GATA6-over expressed Calu-3 cells. Cells were infected using MOI=0.002 for 48 hours. Data were analyzed by two-tailed student's t-test. Shown are means ± SD.

Supplementary Table 1

Supplementary Table 1. CRISPR Editing Efficiencies in Calu-3 Cells as determined by Real-Time PCR.

Target	% of expression relative to Non-Targeted cells
CTDSPL2	29
STX4	51
RER	30
AP1G1	22
SLC35F4	50
CHMP2B	14
EFHB	40
SETDB1	13
IRF6	10
DUSP7	36
HUS	31
EP300	19
CUL5	16
GATA6	38
ACE2	15

Supplementary Table 2

Supplementary Table 2. Oligonucleotide Sequences Used in This Study.

primer	Seq 5-3	
P5 0 nt stagger	AATGATACGGCGACCACCGAGATCTACACTCTTTCCCTACACGACGCTCTTCCGAT CTTTGTGGAAAGGACGAAACACCG	Screens sequencing
P5 1nt stagger	AATGATACGGCGACCACCGAGATCTACACTCTTTCCCTACACGACGCTCTTCCGAT CTCTTGTGGAAAGGACGAAACACCG	
P5 2 nt stagger	AATGATACGGCGACCACCGAGATCTACACTCTTTCCCTACACGACGCTCTTCCGAT CTGCTTGTGGAAAGGACGAAACACCG	
P5 3 nt stagger	AATGATACGGCGACCACCGAGATCTACACTCTTTCCCTACACGACG CTCTTCCGATCTAGCTTGTGGAAAGGACGAAACACCG	
P5 4 nt stagger	AATGATACGGCGACCACCGAGATCTACACTCTTTCCCTACACGACGCTCTTCCGAT CTCAACTTGTGGAAAGGACGAAACACCG	
P5 5 nt stagger	AATGATACGGCGACCACCGAGATCTACACTCTTTCCCTACACGACGCTCTTCCGAT CTTGCACCTTGTGGAAAGGACGAAACACCG	
P5 6 nt stagger	AATGATACGGCGACCACCGAGATCTACACTCTTTCCCTACACGACGCTCTTCCGAT CTACGCAACTTGTGGAAAGGACGAAACACCG	
P5 7 nt stagger	AATGATACGGCGACCACCGAGATCTACACTCTTTCCCTACACGACGCTCTTCCGAT CTGAAGACCCTTGTGGAAAGGACGAAACACCG	
P7 A01	CAAGCAGAAGACGGCATACGAGATCGGTTCAAGTGACTGGAGTTCAGACGTGTG CTCTTCCGATCTCCAATTCCCACTCCTTTCAAGACCT	
P7 A02	CAAGCAGAAGACGGCATACGAGATGCTGGATTGTGACTGGAGTTCAGACGTGTG CTCTTCCGATCTCCAATTCCCACTCCTTTCAAGACCT	
P7 A03	CAAGCAGAAGACGGCATACGAGATTAACTCGGGTGACTGGAGTTCAGACGTGTG CTCTTCCGATCTCCAATTCCCACTCCTTTCAAGACCT	
P7 A04	CAAGCAGAAGACGGCATACGAGATTAACAGTTGTGACTGGAGTTCAGACGTGTG CTCTTCCGATCTCCAATTCCCACTCCTTTCAAGACCT	
P7 A05	CAAGCAGAAGACGGCATACGAGATATACTCAAGTGACTGGAGTTCAGACGTGTG CTCTTCCGATCTCCAATTCCCACTCCTTTCAAGACCT	
P7 A06	CAAGCAGAAGACGGCATACGAGATGCTGAGAAGTGACTGGAGTTCAGACGTGTG CTCTTCCGATCTCCAATTCCCACTCCTTTCAAGACCT	
P7 A07	CAAGCAGAAGACGGCATACGAGATATTGGAGGGTGACTGGAGTTCAGACGTGTG CTCTTCCGATCTCCAATTCCCACTCCTTTCAAGACCT	
P7 A08	CAAGCAGAAGACGGCATACGAGATTAGTCTAAGTGACTGGAGTTCAGACGTGTG CTCTTCCGATCTCCAATTCCCACTCCTTTCAAGACCT	
PRORY F	CACCGTGCTGCTGTGGACGATATCG	Cloning of pLentiCRISP RV2
PRORY R	AAACCGATATCGTCCACAGCAGCAc	
EXTL2 F	CACCGCGTAGGGAAATAAAATCCCA	
EXTL2 R	AAACTGGGATTTTATTTCCCTACGc	
AP1G1 F	CACCGGTATGCACCTTCCAAACGA	
AP1G1_R	AAACTCGTTTGGAAGGTGCATACC	
DUSP7 F	CACCGACGACTCGAAGAGCTCGTG	
DUSP7_R	AAACCACGAGCTCTTCGAGTCGTC	
SLC35F4 F	CACCGATTCTTGAACTTCAAAACCA	
SLC35F4_R	AAACTGGTTTTGAAGTTCAAGAATc	
PRR18 F	CACCGCTGCCTGAATCTCACCCCCG	
PRR18_R	AAACCGGGGGTGAGATTCAGGCAGc	
IRF6 F	CACCGCAGGGCTCGATCATTAACCC	

IRF6_R	AAACGGGTTAATGATCGAGCCCTGc	
CHMP2B F	CACCGAACAATGCAGAATTTCCAGA	
CHMP2B_R	AAACTCTGGAAATTCTGCATTGTTc	
EFHB F	CACCGAAAAGAAGATTCCCGATGCG	
EFHB_R	AAACCGCATCGGGAATCTTCTTTTc	
CTDSPL2 F	CACCGTTATTCATCAGCCCACGCGG	
CTDSPL2 R	AAACCGCGTGGGCTGATGAATAAC	
KREMEN2_g1_F	CACCGAAACAGATCTGGTCACAGT	
KREMEN2_g2_F	CACCGCCCACCCGCAGTAACCCAGA	
KREMEN2_g3_F	CACCGCTTCCCGGACGAGTACGGGC	
KREMEN2_g4_F	CACCGTGAATGGGGCTGACTACCG	
KREMEN2_g1_R	AAACACTGTGACCAGATCTGTTTC	
KREMEN2_g2_R	AAACTCTGGGTTACTGCGGGTGGGc	
KREMEN2_g3_R	AAACGCCCGTACTCGTCCGGGAAGc	
KREMEN2_g4_R	AAACCGGTAGTCAGCCCCATTCAC	
SETDB1_g1_F	CACCGAAGGAAAGAGTCTACTGTCG	
SETDB1_g2_F	CACCGAGATGTGAGTGGATCTATCG	
SETDB1_g3_F	CACCGCCTTACCTGAATCAATACTG	
SETDB1_g4_F	CACCGTTATCTATAAGACACCTTG	
SETDB1_g1_R	AAACCGACAGTAGACTCTTTCCTTc	
SETDB1_g2_R	AAACCGATAGATCCACTCACATCTc	
SETDB1_g3_R	AAACCAGTATTGATTCAGGTAAGGc	
SETDB1_g4_R	AAACCAAGGTGTCTTATAGATAAC	
LARS2_g1_F	CACCGCCATCAGCGACACCATAGCA	
LARS2_g1_R	AAACTGCTATGGTGTCGCTGATGGC	
LARS2_g2_F	CACCGCCAGCCACAGACTCCTACAT	
LARS2_g2_R	AAACATGTAGGAGTCTGTGGCTGGC	
HUS1_g1_F	CACCGAAGGACTTACAAGAACCGG	
HUS1_g1_R	AAACCCGGTTCTTGTAAGTCCTTC	
HUS1_g2_F	CACCGTGTGACAAGCTGGCTAATGG	
HUS1_g2_R	AAACCCATTAGCCAGCTTGTCACAC	
STX4_g1_F	CACCGCGATGAGATCAAACAGCTG	
STX4_g1_R	AAACCAGCTGTTTGATCTCATCGC	
STX4_g2_F	CACCGCCTTAAATGAGATCTCGGCC	
STX4_g2_R	AAACGGCCGAGATCTCATTTAAGGC	
MMP23B__g1_F	CACCGCCTTCTTCCCCCCGCACGG	
MMP23B_g1_R	AAACCCGTGCGGGGGGAAGAAGGC	
MMP23B_g2_F	CACCGCACGTCGCTCCACATGCGGA	
MMP23B_g2_R	AAACTCCGCATGTGGAGCGACGTGC	
EP300 _g1_F	CACCGATGGTGAACCATAAGGATTG	
EP300 _g1_R	AAACCAATCCTTATGGTTCACCATC	
EP300 _g2_F	CACCGTGGCACGAAGATATTACTC	
EP300 _g2_R	AAACGAGTAATATCTTCGTGCCAC	

CUL5 _g1_F	CACCGATTGGAGTAAGAGAATCCTA	
CUL5 _g1_R	AAACTAGGATTCTCTTACTCCAATC	
CUL5_g2_F	CAACGAGGAACATATCATTAGTGC	
CUL5_g2_R	AAACGCACTAATGATATGTTCCTC	
RER1__g1_F	CACCGCGTCCATGGGAAACCTTCGG	
RER1_g1_R	AAACCCGAAGGTTTCCCATGGACGC	
RER1_g2_F	CACCGCTACCCACCAAACAGAACG	
RER1_g2_R	AAACCGTTCTGTTTGGTGGGTAGC	
ACE2 sgRNA F	CACCGATGAGCACCATCTACAGTAC	
ACE2 sgRNA R	AAACGTACTGTAGATGGTGCTCATC	
GATA6 sgRNA F	CACCGCAGTACAGCTCGCTGTCGGC	
GATA6 sgRNA R	AAACGCCGACAGCGAGCTGTACTGC	
seq lenti-crisper V2 guide	GACTATCATATGCTTACCGT	Sequencing pLentiCRISPRV2
CUL5 RT F	GGGATGATAAAGGCCCAGCA	Real-Time PCR
CUL5 RT R	GCCGTATCATCTTGATGGCTC	
EP300 RT F	ACCAGGAATGACTTCTAGTTTGA	
EP300 RT R	GTGCTGAAGAGGAGGGGTTT	
SETDB1 RT F	ATCTGAGGTGGCTCACGTTG	
SETDB1 RT R	GCCGTGTAGAGCCTCGATAG	
KREMEN2 RT F	GCTCATCTCCGCTCTCTGAC	
KREMEN2 RT R	AGCACAGCATCTCAGGTGTC	
IRF6 RT F	GGTAGCGACGGGTGATCTTC	
IRF6 RT R	CCAGGCCTTTGCTCAATCTG	
EXTL1 RT F	AACAGATGCCATCCTCAGCC	
EXTL1 RT R	GCCACACCAGAAAGGCAAAG	
PRORY RT F	CTGTTACTGGCTGGGGAGTG	
PRORY RT R	TGGTCGAGGAGAAGTCCGAT	
AP1G1 RT F	ACATGCTTGCGCATTTCAGA	
AP1G1 RT R	TCCGGACATGATGAGGTTCT	
DUSP7 RT F	CCAGGAGACTTGCACCCATT	
DUSP7 RT R	GTGAACAGAGCCTTACCCCC	
PRR18 RT F	AAAGCGGAAGGGTAGGCATC	
PRR18 RT R	AATGTAATCAGCCGAGCGGT	
SLC35F4 RT F	TCTATGGATGTCAAGGCGGC	
SLC35F4 RT R	GCCATAGTAGCCGGTGATCC	
LARS2 RT F	CCGGCTTATGATTTGAGGGC	
LARS RT R	GGCCCACCATTTAGCTGTCT	
EFHB RT F	TTCTTGGCGTTAAGCAATTCTTCT	
EFHB RT R	CAATGTCCACCTCCACAGGAAG	
CTDSPL2 RT F	GGGGAAGAGTGGACTGGGAA	
CTDSPL2 RT R	TCTCGAGATCCAGCGAGACA	
CHMP2B RT F	AGAAAACCGTGGATGGAATTAGA	

CHMP2B RT R	AGATGCACAAGTTGTTTGGCT	
HUS1 RT F	AGGAACGGAAGGAAGCAGTG	
HUS1 RT R	TTATCAGGGCTGATGCGGAG	
MMP23B RT F	GCAGCGCAGGGAGACG	
MMP23B RT R	GGCTGGAGTCAGCGTGTAG	
STX4 RT F	GGAGAGTGAGTGAAACCCCG	
STX4 RT R	CCCCACCTCAACAAACCACT	
RER RT F	CGGAGCTGCGAGTTACAGAA	
RER RT R	CACCACCGAAGGTTTCCCAT	
ACE2 RT F	GGTCTTCTGTCACCCGATTT	
ACE2 RT R	CATCCACCTCCACTTCTCTAAC	
GATA6 RT F	CACTACCTGTGCAACGCCTG	
GATA6 RT R	GCCGTGATGAAGGCACGC	
GAPDH RT F	TGCACCACCAACTGCTTAGC	
GAPDH RT R	GGCATGGACTGTGGTCATGAG	
hs.Ri.GATA6.13.1-SEQ1	rGrUrCrCrArArArArUrCrArUrGrUrGrCrUrUrCrUrUrCrUGA	siRNA
hs.Ri.GATA6.13.1-SEQ2	rUrCrArGrArArGrArArGrCrArCrArUrGrArUrUrUrUrGrGrArCrArA	
hs.Ri.GATA6.13.2-SEQ1	rCrArArArGrArCrUrUrGrCrUrCrUrGrGrUrArArUrArGrCAA	
hs.Ri.GATA6.13.2-SEQ2	rUrUrGrCrUrArUrUrArCrCrArGrArGrCrArArGrUrCrUrUrUrGrArU	
hs.Ri.GATA6.13.3-SEQ1	rArArArCrGrArArArArCrCrUrArArGrArArCrArUrArArATA	
hs.Ri.GATA6.13.3-SEQ2	rUrArUrUrUrArUrGrUrUrCrUrUrArGrGrUrUrUrUrCrGrUrUrUrCrC	
ACE-2-F	GGTCTTCTGTCACCCGATTT	Clinical samples Real-time PCR
ACE-2-R	CATCCACCTCCACTTCTCTAAC	
ACE-2-P	CAATGAAACAGAAATAAACTTCTTGCTCAACAA	
GATA-6-F	AGAAGCGCGTGCCTTCATCACGGC	
GATA-6-R	TTTCAGCGCCATTTGGTGGT	
GATA-6-P	CTTGGATTGTCCTGTGCCAACTGTCACAC	
GATA6 prom1 5IRD700 F	5'/5IRD700/CCATACAAACACCAACATTATCTTCATGATCCCTAGTTCT	EMSA
GATA6 prom1 5IRD700 R	5'/5IRD700/AGAACTAGGGATCATGAAGATAATGTTGGTGTTTGTATGG	
GATA6 prom2 5IRD700 F	5'/5IRD700/GTAAAATTATAACATTTTCCGTGTATCTTTAACAGCTTTCTAGGA	
GATA6 prom2 5IRD700 R	5'/5IRD700/TCCTAGAAAGCTGTTAAAGATACACGGAAAATGTTATAATTTTAC	
GATA6 prom1 F	CCATACAAACACCAACATTATCTTCATGATCCCTAGTTCT	EMSA-competitor oligos
GATA6 prom1 R	AGAACTAGGGATCATGAAGATAATGTTGGTGTTTGTATGG	
GATA6 prom2 F	GTAAAATTATAACATTTTCCGTGTATCTTTAACAGCTTTCTAGGA	
GATA6 prom2 R	TCCTAGAAAGCTGTTAAAGATACACGGAAAATGTTATAATTTTAC	

Supplementary Figure 3 source data

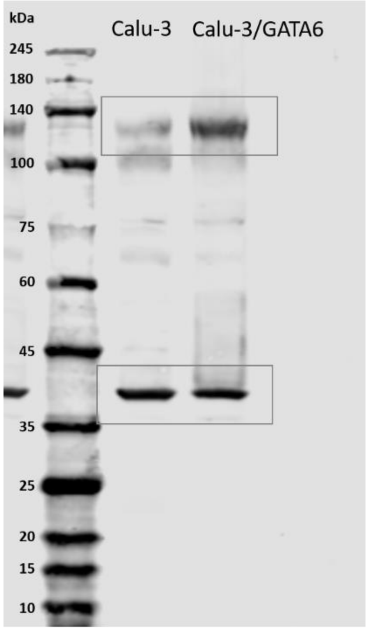

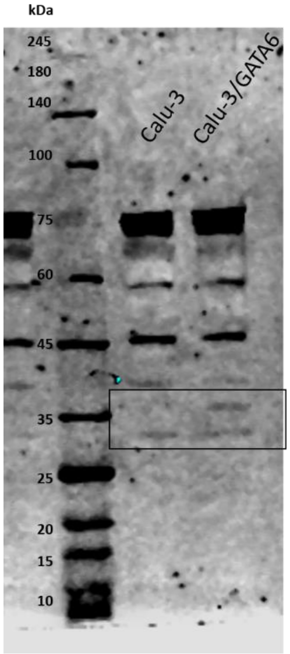

Supplement: Supplementary file 1 — Supplementary Information [file 41467_2022_29896_MOESM1_ESM.pdf]
