# Supplementary material for: Genome-wide CRISPR screens identify GATA6 as a proviral host factor for SARS-CoV-2 via modulation of ACE2
Source: Nat Commun. 2022 Apr 25;13:2237. doi: 10.1038/s41467-022-29896-z (PMC9039069; doi:10.1038/s41467-022-29896-z)
Supplement: Supplementary file 3 — Description of Additional Supplementary [file 41467_2022_29896_MOESM3_ESM.docx]

Description of Additional Supplementary Files

File name: Supplementary Data 1

Description: CRISPR Screen Results: MaGECK Output for Gene Enrichment Analysis of SARS-CoV-2 Host Factor Screens.

File name: Supplementary Data 2

Description: CRISPR Screen Results: MaGECK Output for gRNA Enrichment Analysis of SARS-CoV-2 Host Factor Screens.

File name: Supplementary Data 3

Description: CRISPR Screen Results: MaGECK Output for Gene Enrichment Analysis of Viability screens.
