# Supplementary material for: Genome-wide CRISPR screens identify GATA6 as a proviral host factor for SARS-CoV-2 via modulation of ACE2
Source: Nat Commun. 2022 Apr 25;13:2237. doi: 10.1038/s41467-022-29896-z (PMC9039069; doi:10.1038/s41467-022-29896-z)
Supplement: Supplementary file 7 — Reporting summary [file 41467_2022_29896_MOESM7_ESM.pdf]

nature portfolio

Corresponding author(s): Adi Bercovich-Kinori

Last updated by author(s): Mar 27 2022

Reporting Summary

Nature Portfolio wishes to improve the reproducibility of the work that we publish. This form provides structure for consistency and transparency in reporting. For further information on Nature Portfolio policies, see our Editorial Policies and the Editorial Policy Checklist.

Statistics

For all statistical analyses, confirm that the following items are present in the figure legend, table legend, main text, or Methods section.

n/a	Confirmed	
☐	☒	The exact sample size (n) for each experimental group/condition, given as a discrete number and unit of measurement
☐	☒	A statement on whether measurements were taken from distinct samples or whether the same sample was measured repeatedly
☐	☒	The statistical test(s) used AND whether they are one- or two-sided<br>*Only common tests should be described solely by name; describe more complex techniques in the Methods section.*
☐	☒	A description of all covariates tested
☐	☒	A description of any assumptions or corrections, such as tests of normality and adjustment for multiple comparisons
☐	☒	A full description of the statistical parameters including central tendency (e.g. means) or other basic estimates (e.g. regression coefficient) AND variation (e.g. standard deviation) or associated estimates of uncertainty (e.g. confidence intervals)
☐	☒	For null hypothesis testing, the test statistic (e.g. F, t, r) with confidence intervals, effect sizes, degrees of freedom and P value noted<br>*Give P values as exact values whenever suitable.*
☒	☐	For Bayesian analysis, information on the choice of priors and Markov chain Monte Carlo settings
☒	☐	For hierarchical and complex designs, identification of the appropriate level for tests and full reporting of outcomes
☐	☒	Estimates of effect sizes (e.g. Cohen's d, Pearson's r), indicating how they were calculated

Our web collection on statistics for biologists contains articles on many of the points above.

Software and code

Policy information about availability of computer code

Data collection	*MiSeq Reporter Software 2.5 (Illumina), Primer Express™ Software v3.0.1*
Data analysis	MAGeCK was used to analyze the data using the robust rank aggregation (RRA) algorithm v0.5.6. For gene set enrichment analysis GSEA version 4.1 was used with GO biological process (c5.bp) from MSigDB version 7.4. STRING v11 was used for protein-protein interaction network analysis using default parameters. Image Studio™ Software v5.2 (Licor) was used for Western-blot analysis.The STRING network was imported into Cytoscape v3.8.2. GraphPad Prism v9.2 .

For manuscripts utilizing custom algorithms or software that are central to the research but not yet described in published literature, software must be made available to editors and reviewers. We strongly encourage code deposition in a community repository (e.g. GitHub). See the Nature Portfolio guidelines for submitting code & software for further information.

Data

Policy information about availability of data

All manuscripts must include a data availability statement. This statement should provide the following information, where applicable:

- Accession codes, unique identifiers, or web links for publicly available datasets
- A description of any restrictions on data availability
- For clinical datasets or third party data, please ensure that the statement adheres to our policy

All data generated or analysed during this study are included in this published article (and its supplementary information files).
The sgRNA count raw data generated in this study have been deposited in Gene Expression Omnibus under accession code GSE197962 [https://www.ncbi.nlm.nih.gov/geo/query/acc.cgi?acc=GSE197962]. The processed screen results generated in this study are provided in tables 1-3. Calu3 expression data analysed in this study are available from Gene Expression Omnibus with the accession code GSE162323 [https://www.ncbi.nlm.nih.gov/geo/query/acc.cgi?acc=GSE162323]. Vero-E6 expression data analysed in this study are available from Gene Expression Omnibus with the accession code GSE149973 [https://www.ncbi.nlm.nih.gov/geo/query/acc.cgi?acc=GSE149973]. A549 expression data analysed in this study are available from Gene Expression Omnibus with the accession code GSE82232 [https://www.ncbi.nlm.nih.gov/geo/query/acc.cgi?acc=GSE82232].

Field-specific reporting

Please select the one below that is the best fit for your research. If you are not sure, read the appropriate sections before making your selection.

☒ Life sciences ☐ Behavioural & social sciences ☐ Ecological, evolutionary & environmental sciences

For a reference copy of the document with all sections, see nature.com/documents/nr-reporting-summary-flat.pdf

Life sciences study design

All studies must disclose on these points even when the disclosure is negative.

Sample size	Number of cells in each screen was determined experimentally to be sufficient for the representation of each sgRNA into ~400 unique cells.For in-vitro experiments, no sample size calculation was performed. Sample size is chosen based on the standard of the corresponding field which allows detection of RNA or protein level measured (Optimized sgRNA design to maximize activity and minimize off-target effects of CRISPR-Cas9 . Doench JG. et al. Nat Biotechnol. 2016 Jan 18. doi: 10.1038/nbt.3437. 10.1038/nbt.3437 PubMed 26780180).
Data exclusions	No data were excluded
Replication	Screens and in-vitro experiments were preformed with at least 2 replicates, and with at least 2 biologically independent repeats. All experiment were successfully replicated independently
Randomization	Tissue culture grown cells were randomly assigned treatments
Blinding	Initial sequencing-based analysis of the screens and IFA experiments were performed blinded to the researcher. Researchers performing measurement and data analysis of in vitro assays were not blinded to experimental group due to impracticality of setting up and performing the assays in blinded fashion. However results of all of these experiments were measured via quantitative metrics, reducing risk of bias in the results

Reporting for specific materials, systems and methods

We require information from authors about some types of materials, experimental systems and methods used in many studies. Here, indicate whether each material, system or method listed is relevant to your study. If you are not sure if a list item applies to your research, read the appropriate section before selecting a response.

Materials & experimental systems

n/a	Involved in the study	
☐	☒	Antibodies
☐	☒	Eukaryotic cell lines
☒	☐	Palaeontology and archaeology
☒	☐	Animals and other organisms
☒	☐	Human research participants
☒	☐	Clinical data
☒	☐	Dual use research of concern

Methods

n/a	Involved in the study	
☒	☐	ChIP-seq
☒	☐	Flow cytometry
☒	☐	MRI-based neuroimaging

Antibodies

Antibodies used

αGATA6 (1:500,Abcam ab175349),(1:500, Abcam ab22600) αGAPDH (1:2000,Cell Signaling 14C10), αACE2 (1:1000, Sino Biological #10108-T60),Hyperimmune Rabbit serum from intervenous (i.v) SARS-CoV-2 infected Rabbits. IRDye® 800CW conjugated Goat anti-Rabbit (1:20,000, Licor, P/N 926-32211)

Validation

αGATA6 ,Abcam ab175349- "Rabbit polyclonal to Gata6. Suitable for: ICC/IF, IHC-P, WB. Reacts with: Mouse, Human. Isotype: IgG". Refs: Liu J et al. Gata4 regulates hedgehog signaling and Gata6 expression for outflow tract development. PLoS Genet 15: e1007711 (2019). Ye B et al. LncKdm2b controls self-renewal of embryonic stem cells via activating expression of transcription factor Zbtb3. EMBO J 37:N/A (2018) .
αGATA6 Abcam, ab22600- "Rabbit polyclonal to Gata6. Suitable for: WB, Reacts with:Mouse. Isotype:IgG" Refs:Cao Y et al. HYDIN loss-of-function inhibits GATA4 expression and enhances atrial septal defect risk. Mech Dev 162:103611 (Li Y et al. Derivation of porcine extraembryonic endoderm-like cells from blastocysts. Cell Prolif 53: e12782 .(2020 2020)) .
αGAPDH-Cell Signaling 14C10-nti-GAPDH Antibody is a mouse monoclonal IgG1. Suitable for: WB-Western Blot IP-Immunoprecipitation IHC-Immunohistochemistry ChIP-Chromatin Immunoprecipitation IF-Immunofluorescence F-Flow Cytometry E-P-ELISA-Peptide. GAPDH antibody, cited in 2,210 publications" Refs: Lin, L.|Yang, S.|Xiao, Z.|Hong, P.|Sun, . S.|Zhou, C.|Qian, ZJ. et al. 2021. Mar Drugs. 19
αACE2-Sino Biological #10108-T60- Rabbit Polyclonal. Validated applications: ELISA, IHC-P, Species reactivity Reacts with: Human, Specificity Human ACE2, Immunogen Recombinant Human ACE2 Protein.Refs: Koitka A, et al. (2008) Angiotensin converting enzyme 2 in the kidney. Clin Exp Pharmacol Physiol. 35(4): 420-5. Raizada MK, et al. (2007) ACE2: a new target for cardiovascular disease therapeutics. J Cardiovasc Pharmacol. 50(2): 112-9. Imai Y, et al. (2007) Angiotensin-converting : enzyme 2 (ACE2) in disease pathogenesis. Circ J. 74(3): 405-10. Turner AJ, et al. (2004) ACE2 from vasopeptidase to SARS virus receptor. Trends Pharmacol Sci. 25(6): 291-4 .
Hyperimmune Rabbit serum from intervenous (i.v) SARS-CoV-2 infected Rabbits. Refs:Yahalom-Ronen, Y., et al., A single .dose of recombinant VSV-ΙΙG-spike vaccine provides protection against SARS-CoV-2 challenge. Nat Commun, 2020 p. 6402. Finkel, Y., Gluck, A., Nachshon, A. et al. SARS-CoV-2 uses a multipronged strategy to impede host protein :(1)11 . (synthesis. Nature 594, 240- 245 (2021
IRDye® 800CW conjugated Goat anti-Rabbit (Licor, P/N 926-32211). "The conjugate has been specifically tested and qualified for Western blot and In-Cell Western™ assay applications". Ref: Tirosh, Osnat et al. "The Transcription and Translation Landscapes during Human Cytomegalovirus Infection Reveal Novel Host-Pathogen Interactions." PLoS pathogens vol. 11,11 e1005288. 24 Nov. 2015, doi:10.1371/journal.ppat.1005288

Eukaryotic cell lines

Policy information about cell lines

Cell line source(s)	HEK293T (ATCC- CRL-3216). Vero-E6 (ATCC- CRL-1586) . Calu-3 cells (ATCC- HTB-55), A549 (ATCC-CCL-185)
Authentication	Cell lines were authenticated by ATCC using STR profiling
Mycoplasma contamination	All cell lines tested negative for mycoplasma contamination
Commonly misidentified lines (See ICLAC register)	None
